# Molybdenum tungsten hydrogen oxide doped with phosphorus for enhanced oxygen/hydrogen evolution reactions

**DOI:** 10.1039/d4ra05023a

**Published:** 2024-09-02

**Authors:** Sana Ullah, Asif Hussain, Muhammad Asim Farid, Shaheen Irfan, Roohul Amin, Ahmed M. Fouda, Atif Nazir, Dehua Hou, Ji-Jun Zou, Shangfeng Du, Muhammad Tahir

**Affiliations:** a School of Chemical Engineering and Technology, Tianjin University Tianjin China jj_zou@tju.edu.cn; b Department of Physics, University of Lahore 53700 Lahore Pakistan; c Department of Chemistry, University of Education Lahore 53700 Lahore Pakistan; d School of Sciences, Tianjin University China; e School of Chemical Engineering, Birmingham University Birmingham UK s.du@bham.ac.uk m.tahir.3@bham.ac.uk; f Department of Physics, University of Education Lahore Punjab 54770 Pakistan; g Chemistry Department, Faculty of Science, King Khalid University P. O. Box 9004 Abha 61413 Saudi Arabia; h Institute of Chemical Sciences, Bahauddin Zakariya University Multan 60800 Pakistan

## Abstract

The development of efficient electrocatalysts for hydrogen and oxygen evolution reactions (HER and OER) is pivotal for advancing cleaner and sustainable fuel production technologies. The conventional electrocatalysts have limited stability and higher overpotentials, and there is demand to explore advanced materials and synthesis methods. In this context, a novel bifunctional electrocatalyst has been devised through the phosphidation of tungsten molybdenum oxide (P-Mo_0.69_W_0.31_H_0.98_O_3_) at relatively low temperatures. This innovative approach aims to enhance the efficiency of HER and OER while minimizing the overpotential values and maintaining higher stability. Specifically, the individual performance of Mo_0.69_W_0.31_H_0.98_O_3_ has been significantly boosted by doping it with phosphorus at a low temperature of 300 °C. This doping process results in a unique morphology for the catalyst, leading to a notable improvement in OER/HER performances. P-Mo_0.69_W_0.31_H_0.98_O_3_ exhibits a potential of 320 mV at 10 mA cm^−2^ in a KOH electrolyte, demonstrating both high activity and long-term stability. Additionally, P-Mo_0.69_W_0.31_H_0.98_O_3_ exhibits commendable HER performance, requiring only 380 mV at 100 mA cm^−2^. This combination of efficient OER and HER performance positions P-Mo_0.69_W_0.31_H_0.98_O_3_ as representing a significant advancement in the field of electrocatalysis, additionally addressing the fundamental gap by providing stable and hybrid catalyst for various electrochemical devices. Given its cost-effectiveness and exceptional activity, P-Mo_0.69_W_0.31_H_0.98_O_3_ holds significant potential for advancing the field of electrocatalysis and contributing to the development of cleaner and sustainable fuel production methods.

## Abstract 2

The development of efficient electrocatalysts for hydrogen and oxygen evolution reactions (HER and OER) is pivotal for advancing cleaner and sustainable fuel production technologies.

## Graphical abstract

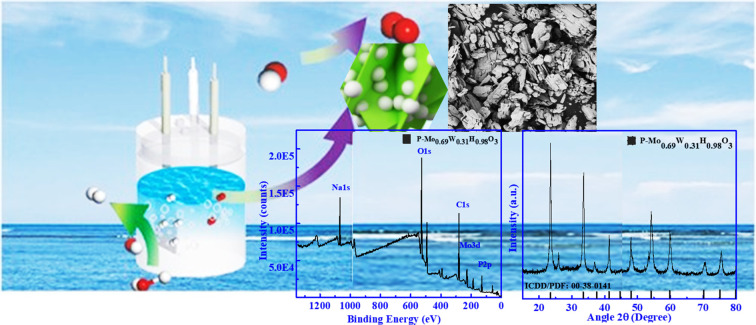

## 1 Introduction

Water splitting, which involves the conversion of water into hydrogen and oxygen through the hydrogen evolution reaction (HER) and the oxygen evolution reaction (OER) with the input of electricity, is a crucial process for producing clean fuels. However, the stability of the electrode and the high overpotentials of OER and HER catalysts present significant challenges for large-scale hydrogen and oxygen production.^1,2^ One of the key challenges in water splitting is the development of efficient and stable catalysts that can drive both HER and OER simultaneously at lower overpotentials, making the process more energy-efficient.^3,4^ This is because the best catalysts for OER typically exhibit poor HER activity, and *vice versa*. Therefore, there is a need for low-cost and stable bifunctional catalysts with the lowest possible overpotentials for both reactions in alkaline exchange membrane water electrolyzers (AEMWEs).^1,5,6^ Addressing this challenge requires innovative approaches in catalyst design and synthesis. Researchers are exploring various strategies, including the development of composite materials, nano-structuring, surface modification,^7^ and doping, to enhance the performance and stability of bifunctional catalysts.^8^ By tailoring the composition, structure, and surface chemistry of catalyst materials, it is possible to optimize their activity for both HER and OER while improving their durability under harsh electrochemical conditions.^1,5,6^

OER is a process used to generate O_2_ through electrochemical oxidation of water, which is a half reaction of WEs and is also involved in the charging process of rechargeable metal–air batteries.^9–12^ As the reverse reaction of ORR, OER also proceeds through multistep proton-coupled electron transfer and is kinetically sluggish. Noble metal (*e.g.*, Ir, Ru)-based materials are placed at the top in terms of their stability in all pH values despite the fair activity towards OER.^13^ However, their high price and scarcity are the major bottleneck for applications. Considerable research has been devoted to searching for alternative materials with better OER activity.^14–21^ Earth-abundant first row (3d) transition-metal-based electrocatalysts, like Co, Ni, Mo, W and Fe -based oxides, are considered as an alternative option for OER because they can often produce O_2_ under mild conditions and modest overpotentials. Since the interaction between different metal ions modulates the electronic structure of each other and optimizes the adsorption/interaction of OER intermediates with catalytic sites, the performance of multi-metal oxides usually outperforms that of the corresponding single-metal oxide, however, there are still lot of gaps to be filled.^11,22–30^

Among the transition metals Mo and W can be employed as important components operating materials for energy devices and energy storage owning to their higher melting point, high conductivity, excellent corrosion resistance and thermal conductivity.^31^ Moreover, tungsten oxide has been used in other applications, including catalysis, organic electronics, and gas sensors.^32^ Additionally, the broad spectrum of WO_*x*_ stoichiometries arises from a dual source, the presence of diverse Magnéli phases and the facile formation of oxygen defect sites, which are included W_32_O_84_, W_3_O_8_, W_18_O_49_, W_20_O_58_, and W_25_O_73_.^33^ The monoclinic WO_2.72_ can be used as a catalyst for its unique crystal structures.^34^ Similarly, Mo and W based catalysts for electrochemical hydrogen production through water splitting is crucial for harnessing intermittent renewable energy sources efficiently. Optimizing electrocatalytic hydrogen evolution reactions (HER) is a key aspect of this technology. While noble metals like platinum (Pt) exhibit exceptional catalytic activity in HER, their scarcity and high costs limit their practical application in electrolyzers. Hence, researchers have been striving to develop cost-effective electrocatalysts using abundant transition metals. This pursuit aims to make hydrogen production *via* water splitting more accessible and sustainable, facilitating the integration of renewable energy into various sectors.^35–37^

A range of studies have explored the potential of molybdenum–tungsten hydrogen oxide for the OER and HER. Tang *et al.*, and Imran *et al.*, both found that nanowire-structured molybdenum–tungsten oxide, when combined with reduced graphene oxide, demonstrated excellent electrocatalytic performance for HER.^38,39^ Li *et al.*, further enhanced this performance by designing a molybdenum oxide-iron, cobalt, copper alloy hybrid catalyst, which showed high efficiency and stability for both HER and OER.^40^ Hatipoglu and his team expanded on this by exploring the electrocatalytic OER/HER of metal-substituted tungsten diboride, which exhibited low overpotential and favorable stability.^41^ These studies collectively highlight the potential of molybdenum–tungsten hydrogen oxide for both OER and HER, and the importance of its structure and composition in achieving high performance. These works motivated us to design molybdenum tungsten hydrogen oxide (Mo_0.69_W_0.31_H_0.98_O_3_) for bifunctional OER and HER catalyst. However, the OER/HER performance is still not very satisfactory therefore we insert phosphorus into the catalyst. Previous studies also suggested that P doping in Mo_0.69_W_0.31_ H_0.98_O_3_ has positive effect of OER and HER activity due to their alteration in the electronic structure and surface chemistry.^42^

Here in this article, incorporating phosphorus into the molybdenum tungsten hydrogen oxide catalyst (Mo_0.69_W_0.31_H_0.98_O_3_) to enhance its bifunctional OER and HER performance is intriguing. The insertion of phosphorus introduces active sites with lower hydrogen adsorption free energy compared to metal sites, suggesting that phosphorus sites play a crucial role in hydrogen evolution and oxidation reactions on the catalyst surface. This indicates that in alkaline solutions, phosphorus atoms can effectively weaken the H–OH bond, facilitating water dissociation and thereby enhancing catalytic activity. The resulting P-Mo_0.69_W_0.31_H_0.98_O_3_ catalyst exhibits high activity for both OER and HER, surpassing the performance of noble metal-based catalysts such as IrO_2_, RuO_2_, and Pt/C. This achievement holds great promise for advancing the field of electrocatalysis, offering a cost-effective and efficient alternative to noble metals in electrolyzers for hydrogen production.

## 2 Experimental

### 2.1 Regents/compounds for synthesis

MoO_3_, WO_3_, NaH_2_PO_2_, IrO_2_ and RuO_2_ were obtained from the J & K chemicals. Absolute ethanol was obtained from Tianjin Guangfu fine chemical research Centre. Nafion solution 5% was purchased from Sigma Aldrich. High pure water was obtained from the UP-water purification system.

### 2.2 Synthesis of P-Mo_0_._69_W_0.31_H_0.98_O_3_

0.5 mg of molybdenum trioxide (MoO_3_) and 0.5 mg of tungsten trioxide (WO_3_) were annelid in the presence of 0.2 mg of sodium hyphophosphide (NaH_2_PO_2_). This mixture of oxide is heated at 350 °C for 3 h in tube furnace with the continues flow of argon gas, the obtained sample was ground and used for electrochemical analysis. For the fabrication of Mo_0.69_W_0.31_H_0.98_O_3_, all the process is same but without the addition of sodium hyphophosphide.

### 2.3 Characterizations

The morphology of P-Mo_0.69_W_0.31_H_0.98_O_3_ was studied by using a field-emission scanning electron microscope (Hitachi S-4800), transmission electron microscopy (TEM) measurements were done on a Tecnai G2 F-20 transmission electron microscope. The XRD (X-ray diffraction) patterns of the prepared samples were recorded by Bruker D8 Focus operating at 45 kV and 45 mA furnished with nickel-filtered Cu Kα radiation in the range of 10–80° of 2*θ*. The chemical state, surface morphology and composition were analyzed with PHI-5000 X-ray photoelectron spectroscopy (XPS) with Al Kα radiation. The high-resolution transition electron microscopy HRTEM images of catalyst are acquired on a Tecnai G2 F20 microscope operating at a voltage of 200 kV to investigate the carbon deposition morphology and structure.

### 2.4 Electrochemical measurement

The electrochemical test is performed by using IVIUMSTAT workstation (IVIUM Technologies BV, Netherlands). Workstation typically consists of three types of electrodes, a working electrode (catalyst on Ni-foam), graphite rod as a counter electrode and Hg/HgO as a reference electrode. The working electrode was prepared by taking 5 mg of the prepared catalyst in 1 ml of ethanol (analytical grade 99%) and 20 to 30 μl of Nafion binder is added. This solution was sonicated for 10 minutes, followed by addition of ink to Ni-foam by a micropipette and dried at 60 °C. All the experiments are carried out in 1 M KOH electrolytes with a scan rate of 1 mV s^−1^. The linear sweep voltammetry (LSV) and all electrode potentials are converted to a reversible hydrogen electrode. The active surface area of the electrocatalyst was attained from double-layer capacitance of 10 to 100 mV s^−1^. A reversible hydrogen electrode (RHE) was used for potential reference and calculated as follows.1
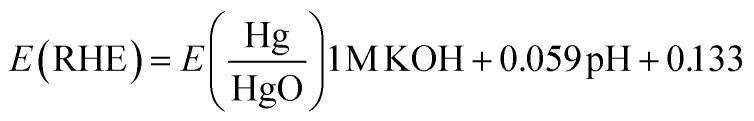


The electrode based on Ni-foam is used for electrochemical measurements, it was washed with dilute HCl and dried for 24 hours at 60 °C. The prepared catalyst is loaded on Ni-foam having size equal to 1 × 1 cm,^2^ dried at room temperature and followed by annealing at 350 °C for 3 h, the scan rate of linear voltammetry (LSV) is set as 1 mV s^−1^.

## 3 Result and discussion

### 3.1 Physicochemical characteristics of P-Mo_0.69_W_0.31_H_0.98_O_3_

**Fig. 1:**
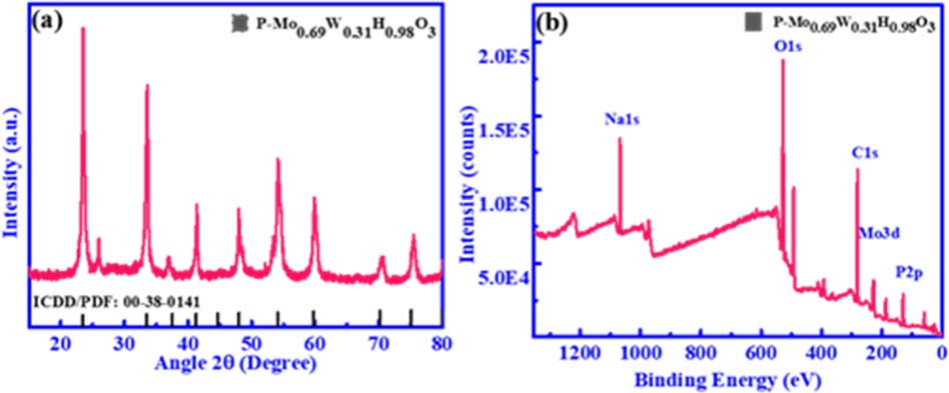
(a) XRD pattern (b) XPS pattern of P-Mo_0.69_W_0.31_H_0.98_O_3_.

**Fig. 2:**
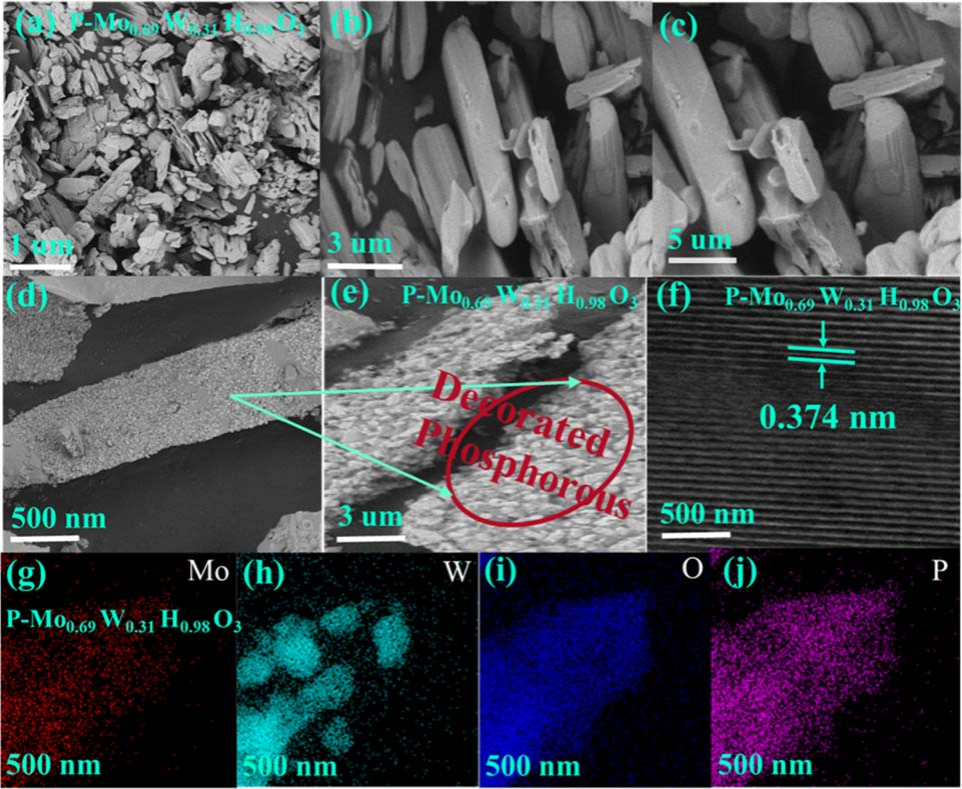
(a–c) SEM images (d–f) HRTEM images (g–j) of elemental mapping of P-Mo_0.69_W_0.31_H_0.98_O_3_.

Structural characterization of as-synthesized products is done by employing XRD. Fig. 1(a) represented XRD patterns of P-Mo_0.69_W_0.31_H_0.98_O_3_. The main Peaks signatures are sharp, which attributed higher crystallinity has been achieved, additionally includes composition determination, structural identification and oxygen excess is presents in P-Mo_0.69_W_0.31_H_0.98_O_3_ composites,^43^ which is significant for enhanced stability and catalytic efficiency. The super positional peaks were not found including, both pure molybdenum oxide and pure tungsten oxide.^44^ The XRD patterns matched well with standard ICDD/PDF No: 00-8-0141. The SEM and TEM images of the product are shown in Fig. 2. It is evident that the resultant product primarily consists of nanorods, showcasing a diverse spectrum of widths ranging from 200 to 400 nm. The length of these nanorods spans from 0.1 to 1.5 μm, as illustrated in Fig. 2(a–c), Furthermore, sodium hypophosphite (NaH_2_PO_2_) is introduced as the phosphorus source to Mo_0.69_W_0.31_H_0.98_O_3_. The phosphorous is deposited on the surface of the synthesized catalyst Mo_0.69_W_0.31_H_0.98_O_3_, as clearly depicted in Fig. 2(d and e). The deposition of ‘P’ is essential for surface properties modification, including electronic structures, surface resistivity, and charge transfer routes. The HRTEM image with well-define layered spacing of 0.374 nm is shown in Fig. 2(f). The spacing distance of 0.374 nm is closely agreement with the (110) planes as given from the standard ICDD/PFD card, corresponding to the (110) orientation of the orthorhombic structure of P-Mo_0.69_W_0.31_H_0.98_O_3_ catalyst. The (110) planes orientation attributes to the ‘p’ integration and potentially induction of catalytic sites. The EDX and element mapping represents the distribution of Molybdenum (Mo), Tungsten (W), Phosphorous (P), and Oxygen (O) along with the molar content of Mo, W, P, and O is approximately 3.15, 57.95, 6.00, and 32.90% respectively. Furthermore, the presence of Mo, W, P, and O are also confirmed by element mapping as mentioned in Fig. 2(g–j). Moreover, elemental weight percentage for P-Mo_0.69_W_0.31_H_0.98_O_3_ is listed in Table 1. The above characterization clearly showed that alloys of molybdenum tungsten hydrogen oxide and phosphorus are well mixed in the form of unique morphology.

**Table 1:** The elemental percentage of P-Mo_0.69_W_0.31_H_0.98_O_3_

Element	Weight %	Atomic %
O k	32.90	79.15
P k	6.00	7.46
Mo k	3.15	1.26
W k	57.95	12.13

**Fig. 3:**
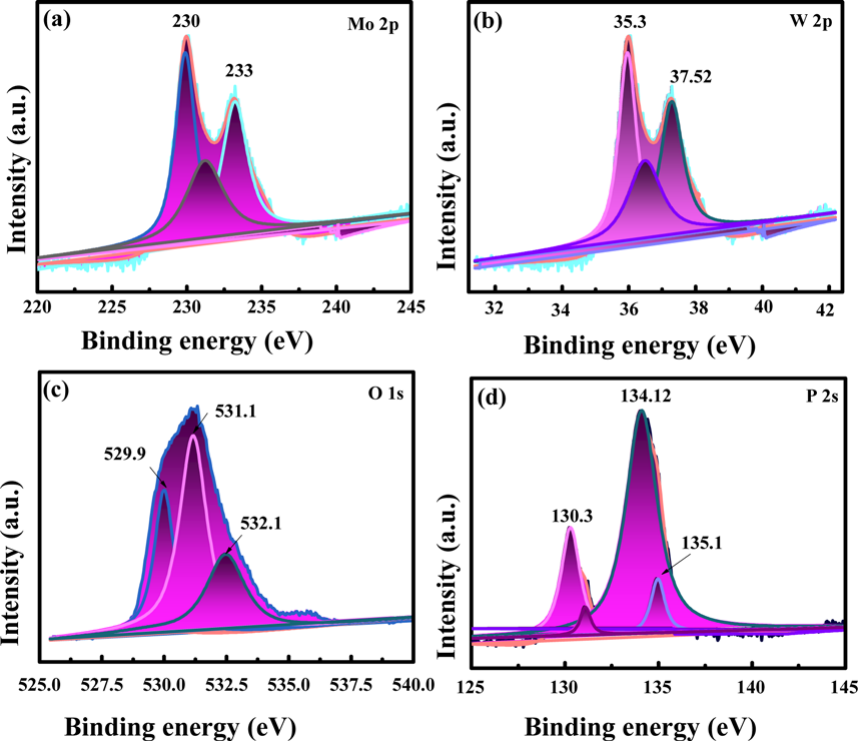
XPS spectra of (a) Mo 2p (b) W 2p (c) O 1s (d) P 2s P-Mo_0.69_W_0.31_H_0.98_O_3_.

The chemical nature and chemical bonding for P-Mo_0.69_W_0.31_H_0.98_O_3_ is investigated by X-ray photoelectron spectroscopy (XPS). Obtained peak signature corresponding to the elements such as Mo, W, P, and O exiting in P-Mo_0.69_W_0.31_H_0.98_O_3_ is shown in Fig. 1b and 3. The presence of these elements exhibits a strong agreement with elemental mapping. Furthermore, Fig. 3(a) represented the Mo spectra for P-Mo_0.69_W_0.31_H_0.98_O_3_, which can be fitted with two peaks at 233.1 eV and another at 236.4 eV energies, which corresponding to Mo 3d_5/2_ and Mo 3d_3/2_, attributed the Mo^+6^ (+6) valence state and significant for OER.^45,46^Fig. 3(b) shows the W spectra in catalyst P-Mo_0.69_W_0.31_H_0.98_O_3_, which can be fitted with three curves at the position of 35.3, and 37.52 eV, which are corresponding W 4f_7/2_ and W 4f_5/2_ and assumed for the variable oxidation states of tungsten (W) respectively and indicates the +6-oxidation state of Tungsten (W) atom. Furthermore, the most common formal oxidation state of tungsten is +6 used for OER, but it exhibits in all oxidation states from −2 to +6. Tungsten typically combines with oxygen to form a yellow tungsten oxide such as WO_3_, which dissolves in aqueous alkaline solutions to form tungstate ions WO^−2^. All the binding energies are assigned to +6 oxidation state because +6 oxidation state is a formal oxidation state of Tungsten.^47–49^ However, a peak at 35.04 eV is related to +5 the oxidation state of the Tungsten (W). However, the higher oxidation states, such as +6 are always presenting oxides as compared to lower oxidation states. The presence of such oxides is relevant to their terrestrial occurrence and biological roles, whereas the middle oxidation states are often associated with metal clusters, while low oxidation states are typically associated with its complexes. The comparison of Tungsten (W) with Molybdenum (Mo) individually showed that both are less efficient towards the electrocatalyst but when combined in the form of phosphidation of molybdenum tungsten hydrogen oxide they bring an efficient catalyst for OER.^49,50^ The phosphidation of bimetallic oxide also provides a great role in the activity of metallic oxide. The phosphorous source resulted in the formation of a strong bond with metal oxides which increased the stability and durability of an electrocatalyst.^48^

Fig. 3(c) represented the O 1s spectrum, which includes three main peaks at 529.5 eV and 531.3 eV and 531.5 eV.^51^ The peak at 531.5 eV is broader than the other two peaks, these broader peaks are associated with surface hydroxyl species and a smaller peak at 529.5 eV is associated with sulfide species. The high resolution of the P 2p spectrum represented a peak at 130.3 eV Fig. 3(d) which denoted the bond between phosphorous and carbon. Moreover, the Peaks at 129 eV and 134 eV are related to the binding energy of P 2p_3/2_ and P 2p_1/2_ respectively. While the other peaks at 134.12 eV and 135.1 eV represented the presence of oxidized phosphate types of species.

### 3.2 Electrocatalytic OER performance in alkaline media

**Fig. 4:**
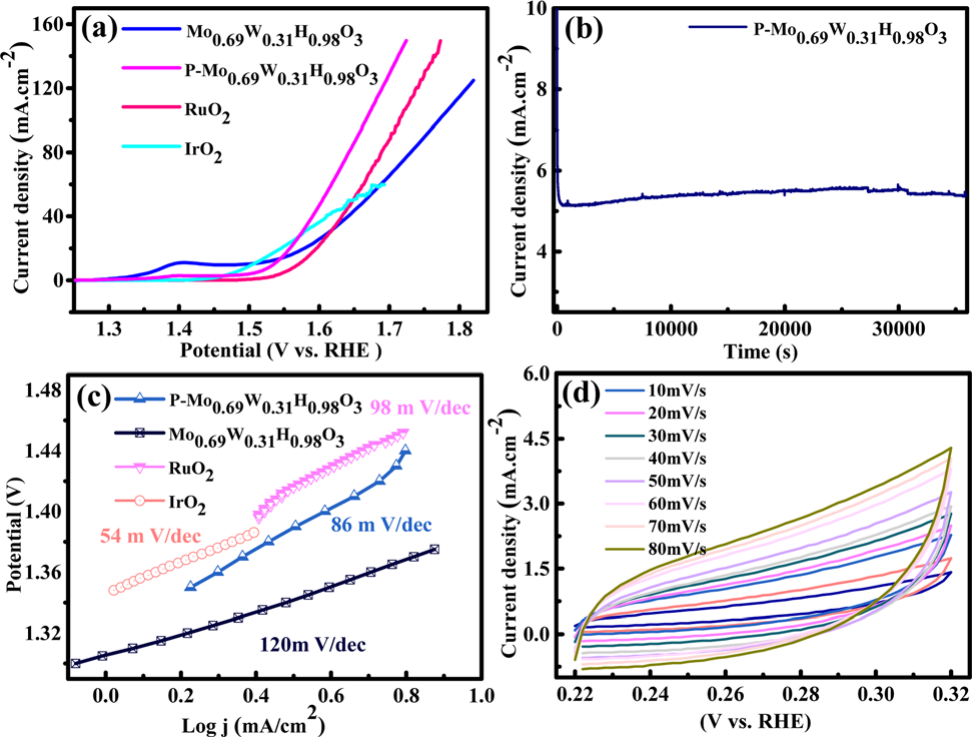
(a) LSV (b) stability (c) Tafel slope (d) CV of P-Mo_0.69_W_0.31_H_0.98_O_3_, Mo_0.69_W_0.31_H_0.98_O_3,_ RuO_2_ and IrO_2_.

We studied the performance of P-Mo_0.69_W_0.31_H_0.98_O_3_ in 1 M KOH solution on Ni-foam (Fig. 4). The OER polarization curves are recorded with a lower scan rate of 1 mV s^−1^. Fig. 4(a) shows the LSV of P-Mo_0.69_W_0.31_H_0.98_O_3_, IrO_2_, and RuO_2_ in alkaline media. P-Mo_0.69_W_0.31_H_0.98_O_3_ showed a potential of 320 mV *versus* RHE at 10 mA cm^−2^ which is better than IrO_2_ (330 mV).

P-Mo_0.69_W_0.31_H_0.98_O_3_ contained an alloy of Mo and W, the alloy of transition metals based electrocatalyst played an active role in the OER. The durability of P-Mo_0.69_W_0.31_H_0.98_O_3_ is investigated by continuous OER performance at static current density, which exhibited a negligible decrease (Fig. 4(b)).

The Tafel slope values for all samples are shown in Fig. 4(c) which described the comparative study of different materials. The Tafel slope of P-Mo_0.69_W_0.31_H_0.98_O_3_ is recorded at 88 mV dec^−1^ while, the Tafel slope value for IrO_2_ and RuO_2_ is 54 mV dec^−1^ and 98 mV dec^−1^ respectively. A smaller value of the Tafel slope is also confirmed for its higher conductivity to support better OER activity. Tafel slope value of less than 120 mV dec^−1^ is more favorable for OER as reported by different research groups.^52^ The cyclic voltammogram (CV) is represented in Fig. 4(d), which is another important factor, which is recorded at various scan rates of (10–100 mV s^−1^). The CV curves represented a complete rectangular performance on each scan rate, which enhanced the double-layer capacitance (*C*_dl_).

### 3.3 Electrocatalytic HER Performance in alkaline medium

**Fig. 5:**
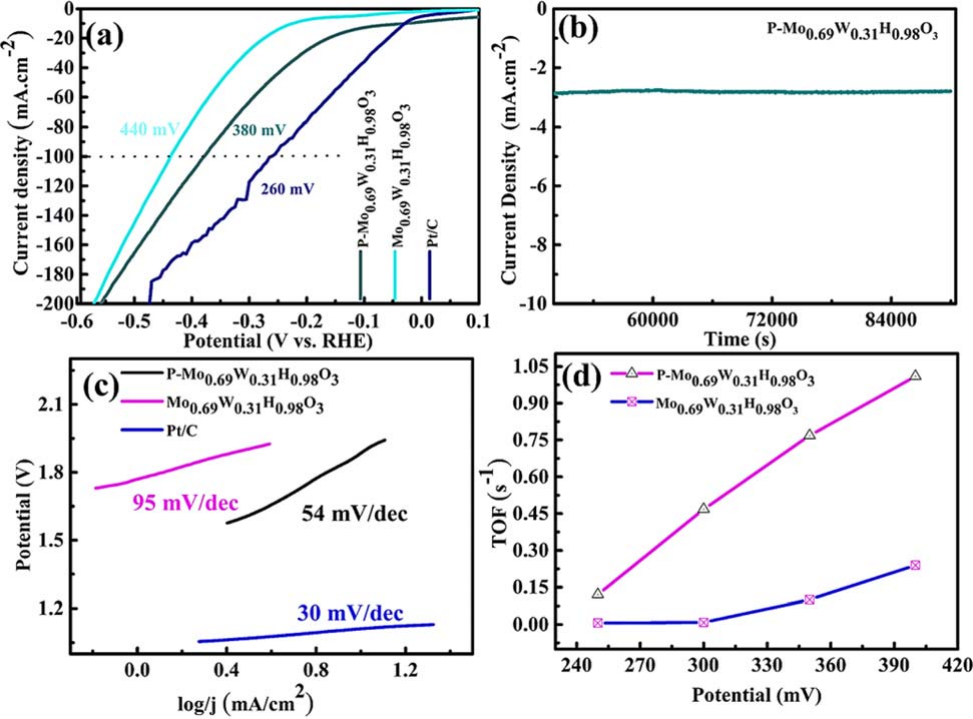
(a) LSV (b) stability (c) Tafel slope (d) and TOF of different catalyst.

The LSV curves of all the samples for HER activity are shown in Fig. 5(a) in alkaline media. The HER activity of P-Mo_0.69_W_0.31_H_0.98_O_3_ is 380 mV at 100 mA cm^−2^ is improved compared with Mo_0.69_W_0.31_H_0.98_O_3_ (440 mV) and less than Pt/C (260 mV). The durability of P-Mo_0.69_W_0.31_H_0.98_O_3_ is investigated by continuous HER performance at static current, which exhibited a negligible decrease (Fig. 5(b)). This represented P-Mo_0.69_W_0.31_H_0.98_O_3_ is highly stable for HER in alkaline media. Moreover, the Tafel slope of P-Mo_0.69_W_0.31_H_0.98_O_3_ is 54 mV dec^−1^ is better as compared to Mo_0.69_W_0.31_H_0.98_O_3_ and Pt/C as can be seen in Fig. 5(c). Similarly, the turnover frequency (TOF) of P-Mo_0.69_W_0.31_H_0.98_O_3_ is recorded as 1.02 which is higher compared with the individual TOF values of P-Mo_0.69_W_0.31_H_0.98_O_3_ (Fig. 5(d)).

**Fig. 6:**
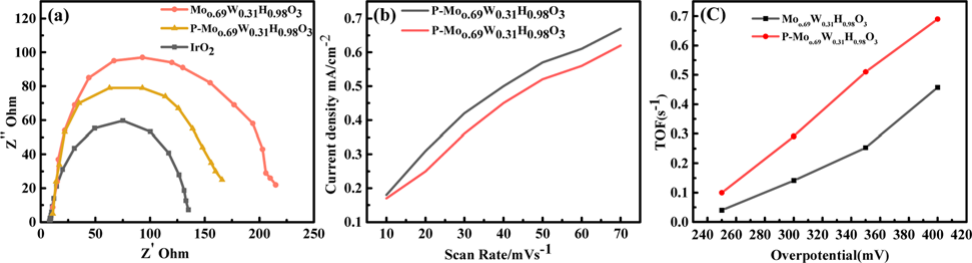
(a) EIS (b) *C*_dl_ (c) and TOF values of an electrocatalyst P-Mo_0.69_W_0.31_H_0.98_O_3_.

The electrochemical impedance spectroscopy (EIS) is a critical factor for the analysis of kinetics through the catalytic process (Fig. 6). The EIS study is related to charge transfer resistance (*R*_tc_). The smaller semicircle of EIS is often related to the fast electron transmission process. The EIS values for P-Mo_0.69_W_0.31_H_0.98_O_3_ showed the resistance of 83 ohm, the smaller *R*_ct_ value is evidence of the superior performance of the catalyst as can be seen through Fig. 6(a). Moreover, the electrical conductivity and the number of active sites are necessary factors for the better performance of electrocatalyst.^53,54^ The enhancement of the conductivity, as well as the increase of active sites of catalysts, will enhance the catalytic activities. The results showed that P-Mo_0.69_W_0.31_H_0.98_O_3_ exhibited superior performance for faster electron transmission and thus leading to a better catalytic activity.

The *C*_dl_ is directly related to the number of active sites of electrocatalyst (Fig. 6(b)). The exchange of current density is also normalized by the double-layer capacitance *C*_dl_ which is directly revealed the more catalytic activity.^15^ It is worth noticing that P-Mo_0.69_W_0.31_H_0.98_O_3_ has a 51.0 mF cm^−2^*C*_dl_ value, which is more significant than all other synthesized samples. The higher value of current density for P-Mo_0.69_W_0.31_H_0.98_O_3_ further proved that variable oxidation states of Mo and W. These different oxidation states are responsible for the presence of more active sites in the catalyst under study.^55^ The turnover frequency (TOF) is another essential factor for the evaluation of better performance of electrocatalyst for OER (Fig. 6(c)).^56,57^ The TOF value of P-Mo_0.69_W_0.31_H_0.98_O_3_ is 0.06 (s^−1^) at potential of 320 mV, which is greater than TOF values observed for RuO_2_ and IrO_2_.

## 4 Conclusion

The phosphidation of molybdenum tungsten hydrogen oxide alloy resulted in the successful synthesis of P-Mo_0.69_W_0.31_H_0.98_O_3_ by employing a straightforward and industrially scalable method. This catalyst has a significant potential as an alternative to noble metal electrocatalyst for OER. The catalyst provided a significant potential for both the reactions OER/HER with long term stability and enhanced catalytic activity in alkaline electrolytes. Moreover, the development of P-Mo_0.69_W_0.31_H_0.98_O_3_ catalyst not only provides a stable and efficient low-cost electrocatalyst for bifunctional catalysis, but it also gives safe and easy idea to apply this electrocatalyst for industrial application.

## Data availability

The data that support the findings of this study are available on request from the corresponding author.

## Author contributions

The manuscript was written through the contributions of all authors. All authors have given approval to the final version of the manuscript.

## Conflicts of interest

There are no conflicts to declare.
